# Establishing an internal quality control method for the stable extraction of nucleic acids of severe acute respiratory syndrome coronavirus 2 and RT‐PCR‐based detection

**DOI:** 10.1002/jcla.23998

**Published:** 2021-10-02

**Authors:** Takahiro Okuyama, Kouki Ohtsuka, Wataru Ogura, Shota Yonetani, Satoko Yamasaki, Hiroyuki Miyagi, Kumiko Sekiguchi, Hiroaki Ohnishi, Takashi Watanabe

**Affiliations:** ^1^ Clinical Laboratory Department Kyorin University Hospital Tokyo Japan; ^2^ Department of Laboratory Medicine Kyorin University School of Medicine Tokyo Japan; ^3^ Department of Clinical Laboratory Technology Faculty of Health Sciences Kyorin University Tokyo Japan

**Keywords:** COVID‐19, quality control, quality control chart, quality control substance, real‐time RT‐PCR, SARS‐CoV‐2

## Abstract

**Background:**

Severe acute respiratory syndrome coronavirus 2 (SARS‐CoV‐2), the causative agent of the coronavirus disease 2019 (COVID‐19), is detected using real‐time RT‐PCR. However, there are limitations pertaining to quality control, particularly with respect to establishing quality control measures for extraction of viral nucleic acids. Here, we investigated the quality control measures for the various processes using an extrinsic quality control substance and quality control charts.

**Methods:**

An extrinsic quality control substance was added to the sample, and then, real‐time RT‐PCR was performed. Samples with negative test results and the corresponding data were analyzed; a quality control chart was created and examined.

**Results:**

Data analysis and the quality control charts indicated that SARS‐CoV‐2 could be reliably detected using real‐time RT‐PCR, even when different nucleic acid extraction methods were used or when different technicians were employed.

**Conclusion:**

With the use of quality control substances, it is possible to achieve quality control throughout the process—from nucleic acid extraction to nucleic acid detection—even upon using varying extraction methods. Further, generating quality control charts would guarantee the stable detection of SARS‐CoV‐2.

## Graphical abstract

Real‐time RT‐PCR is used in many countries to detect SARS‐CoV‐2; however, there is no standardized method for quality control during RT‐PCR‐based detection. Our study showed that the use of a quality control substance (EAV) and quality control charts ensures internal quality control for the extraction of SARS‐CoV‐2 nucleic acid and the subsequent virus detection.

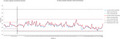

## 1 INTRODUCTION

Coronavirus disease 2019 (COVID‐19) is an infectious disease caused by severe acute respiratory syndrome coronavirus 2 (SARS‐CoV‐2).^1^ The COVID‐19 outbreak originated in Wuhan City, Hubei Province, China, in December 2019 and reached pandemic proportions by March 2020.^2^ In Japan, the first infection was confirmed in January 2020, and the number of infected people increased rapidly.^3^ COVID‐19 is diagnosed by detecting SARS‐CoV‐2 using real‐time reverse transcription‐polymerase chain reaction (RT‐PCR), as described in the Pathogen Detection Manual 2019‐nCoV of the National Institute of Infectious Diseases.^4^

The use of internal quality control standards greatly influences the accuracy of the test results and is therefore vital in diagnostic facilities. The Pathogen Detection Manual 2019‐nCoV details the guidelines for confirming the positive and negative controls used for the quality control of the nucleic acid detection process.^4^ However, there are no guidelines to ensure the quality control of the nucleic acid extraction process.

The effectiveness of the nucleic acid extraction process is influenced by the extraction efficiency, which depends on parameters such as sampling errors, nucleic acid degradation, and presence of inhibitors. It is additionally influenced by the use of samples with a low viral load, poor quality samples, or samples subjected to other prior treatments.^5^ It is important to prevent false negatives in the subsequent confirmation tests. The global spread of COVID‐19 resulted in an urgent need to hire personnel for conducting inspections at many facilities. This urgency increased the probability of hiring inexperienced personnel for conducting diagnostic tests. In such contexts, it is important to establish a quality control method to ensure accurate diagnostic results; however, no such method is currently available for the detection of SARS‐CoV‐2.^6^

The Lightmix Modular EAV RNA Extraction Control Kit from Roche Diagnostics (Basel, Switzerland; hereafter referred to as the EAV reagent kit) enables the quality control of the nucleic acid extraction process. The EAV reagent kit consists of equine arteritis virus nucleic acid (hereafter referred to as the EAV control) and a primer probe that can detect the viral nucleic acid sequence. The EAV reagent is an external control reagent that can be added to a sample; its amplification confirms that the reaction systems in the nucleic acid extraction and detection steps are functioning properly.^7^ Conventionally, nucleic acids are evaluated using the A_280_ and A_280_/A_260_ metrics. The advantage of using the EAV control is that the accuracy of both the nucleic acid extraction and detection processes can be monitored simultaneously. Reagent kits for viral genes encoding the SARS‐CoV‐2 envelope and nucleocapsid proteins and RNA‐dependent RNA polymerase have been developed by Roche Diagnostics for use with EAV reagents.^8^

This retrospective study evaluated the detection of SARS‐CoV‐2 nucleic acids in clinical samples using the N and N2 assays, as described in the Pathogen Detection Manual 2019‐nCoV, and the accuracy of the nucleic acid extraction and detection processes, using the EAV reagent with the conventional and automatic nucleic acid extraction methods. A quality control chart was created based on the EAV control data obtained in this study.

## 2 MATERIALS AND METHODS

### 2.1 Sampling

This study was approved by the ethics committee of Kyorin University, Tokyo, Japan (R02‐042). Informed consent from the participants was not required. Between March and April 2020, nasopharyngeal swabs were collected from 117 patients with suspected but negative COVID‐19. Among these, samples from 16 patients were subjected to the conventional nucleic acid extraction and those from 101 patients to the automatic nucleic acid extraction. For samples testing positive, PCR reagents, such as DNA polymerase, dNTP, and Mg^2+^ were used for SARS‐CoV‐2 nucleic acid detection, which could affect the reaction system of the EAV control, and thus, such samples were excluded from the study.

### 2.2 Reagents and equipment

The conventional extraction method utilized column extraction with QIAamp Viral RNA Mini Kit (QIAGEN), and the automatic nucleic acid extraction method used the magLEAD 6 gC (Precision System Science Co., Ltd.).

For nucleic acid detection, the *N* gene (N, N2) was amplified using a one‐step real‐time RT‐PCR with the QuantiTect Probe RT‐PCR kit (QIAGEN), as described in the Pathogen Detection Manual 2019‐nCoV. The amplicons were analyzed using the COBAS Z480 PCR Analyzer (Roche Diagnostics).^4^ The primers and probes used in the N and N2 assays were those recommended by the National Institute of Infectious Diseases and purchased from Nihon Gene Research Laboratories Inc. (Sendai) (Table 1).

**TABLE 1:** Primer and probe sequences for severe acute respiratory syndrome coronavirus 2 (SARS‐CoV‐2) detection

Target	Oligonucleotide	Sequence (5′ to 3′)	Concentration
N	N_Foward Primer	CACATTGGCACCCGCAATC	Use 600 nM per reaction
	N_Reverse Primer	GAGGAACGAGAAGAGGCTTG	Use 800 nM per reaction
	N_Probe	FAM‐ACTTCCTCAAGGAACAAACATTGCCA‐BHQ	Use 200 nM per reaction
N2	N2_Foward Primer	AAATTTTGGGGACCAGGAAC	Use 500 nM per reaction
	N2_Reverse Primer	TGGCAGCTGTGTAGGTCAAC	Use 700 nM per reaction
	N2_Probe	FAM‐ATGTCGCGCATTGGCATGGA‐BHQ	Use 200 nM per reaction

### 2.3 Experimental procedures

The test was performed by seven medical technicians. In the conventional and automatic nucleic acid extraction methods, 10 and 6 µl of the EAV control were used, respectively, and nucleic acid amplification and detection were performed using a multiplex PCR in the N and N2 assays.

A volume of 10 µl of the EAV control is recommended by the manufacturer; however, 6 µl was used in the automatic extraction method to maintain the crossing point (Cp) value within the range described by the manufacturer, based on the results of a preliminary examination conducted earlier at the hospital. The thermal cycling conditions were set as follows: reverse transcription at 50℃ for 30 min, followed by initial denaturation at 95℃ for 15 min, and 45 cycles of denaturation at 95℃ for 15 s, and annealing/extension at 60℃ for 60 s.^4^

**FIGURE 1:**
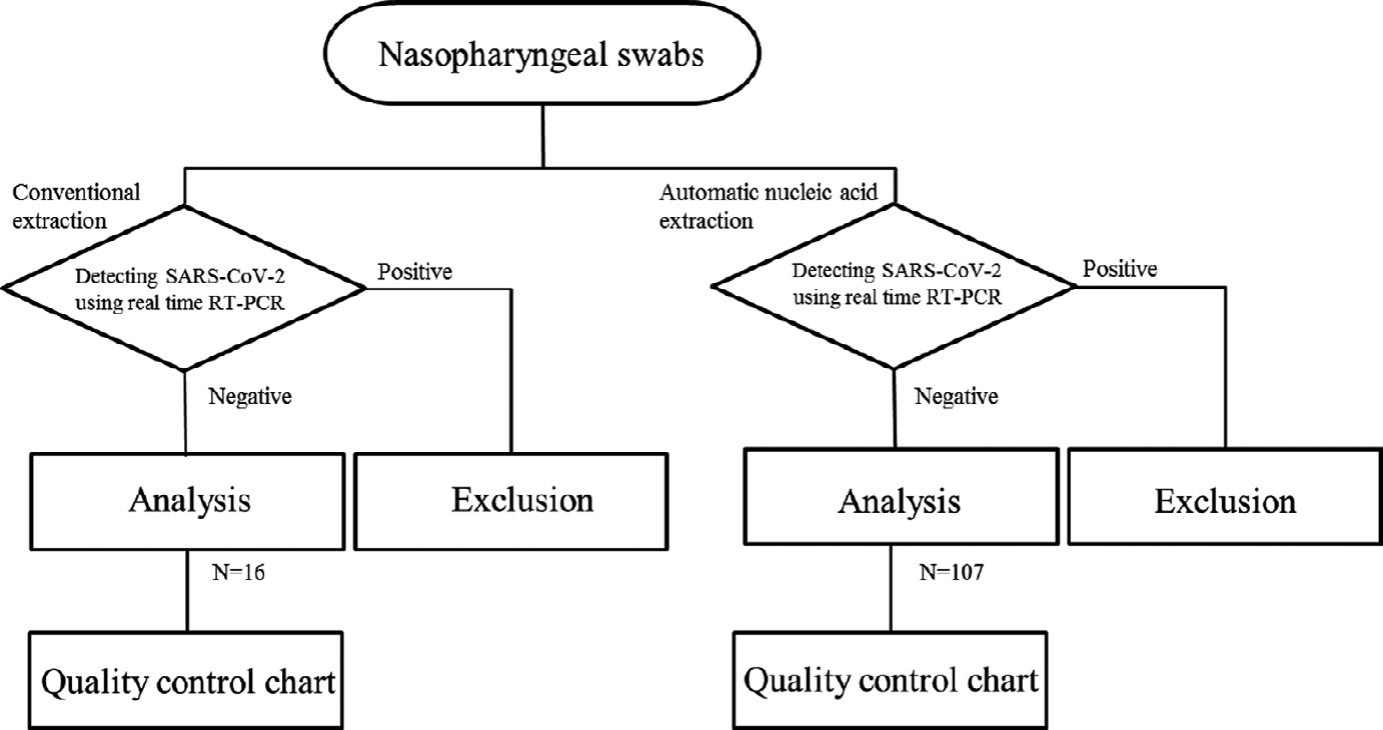
Flowchart of the sampling methods and inclusion and exclusion criteria used in this retrospective study

In the detection analysis, the Cp value was calculated using the automatic judgment of the second derivative method.^4^ Double measurements were performed for all cases to ensure the lowest detection sensitivity (N set: 7 copies, N2 set: 2 copies). If either one of the assays was positive, the test result was defined as positive, with both the values used for generating the control chart.^4^ If either one of the double measurements was positive, the test result was confirmed as positive, and both the Cp values were plotted. The calculated Cp values of the EAV control are presented as the mean ± standard deviation (SD) or coefficient of variation (CV), and the mean + 3SD and mean ‐ 3SD values were used as the upper control limit (UCL) and lower control limit (LCL), respectively (Figure 1).

## 3 RESULTS

### 3.1 CV analysis

The CVs of the Cp values of the samples obtained using conventional or automatic nucleic acid extraction methods were 0.94–2.02% in the N assay and 1.14–1.96% in the N2 assay, which were considered reasonable (Table 2).

**TABLE 2:** Fluctuations in Cp^a^ value in cases where the extraction was performed using the conventional or automatic nucleic acid extractor method

Extraction method	Assay	N	Mean (Cycle)	SD^b^	CV^c^ (%)
Conventional	N	16	28.42	0.27	0.94
	N2		28.52	0.32	1.14
Automatic nucleic acid extractor	N	101	28.45	0.58	2.02
	N2		28.62	0.56	1.96

^a^ Crossing point.

^b^ Standard deviation.

^c^ Coefficient of variation.

### 3.2 X‐bar control charts

**FIGURE 2:**
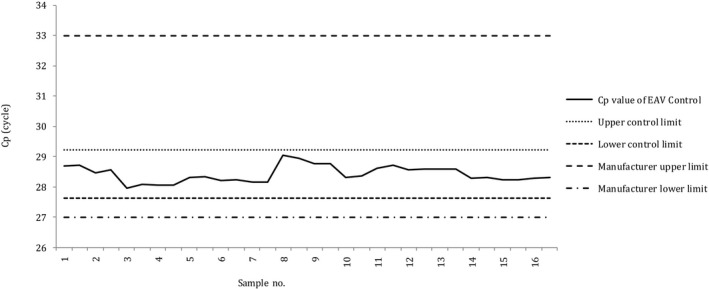
X‐bar control chart of the Cp values of samples processed through the N assay performed using conventional nucleic acid extraction. Cp, crossing point; EAV, equine arteritis virus; LCL, lower control limit; UCL, upper control limit

**FIGURE 3:**
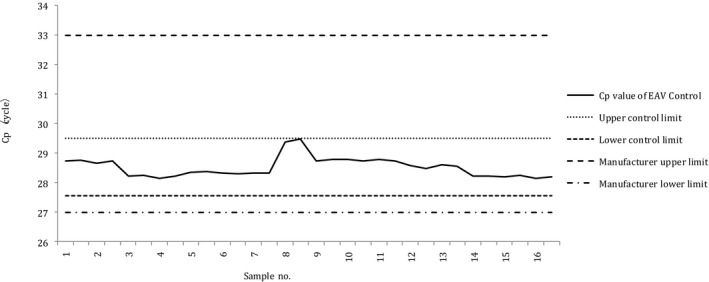
X‐bar control chart of the Cp values of samples processed through the N2 assay performed using conventional nucleic acid extraction. Cp, crossing point; EAV, equine arteritis virus; LCL, lower control limit; UCL, upper control limit

The X‐bar control charts of the Cp values of samples obtained through the N and N2 assays, using the conventional nucleic acid extraction method, are presented in Figures 2 and 3, respectively. In both charts, no cases deviated from the UCL or LCL, and no cases deviated from the recommended range of the EAV reagent (27–33).

**FIGURE 4:**
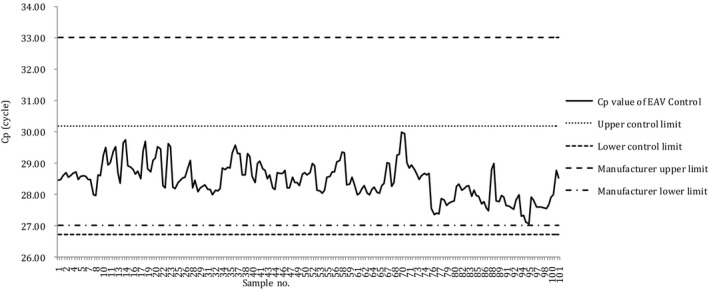
X‐bar control chart of the Cp values of samples processed through the N assay performed using automatic nucleic acid extraction. Cp, crossing point; EAV, equine arteritis virus; LCL, lower control limit; UCL, upper control limit

**FIGURE 5:**
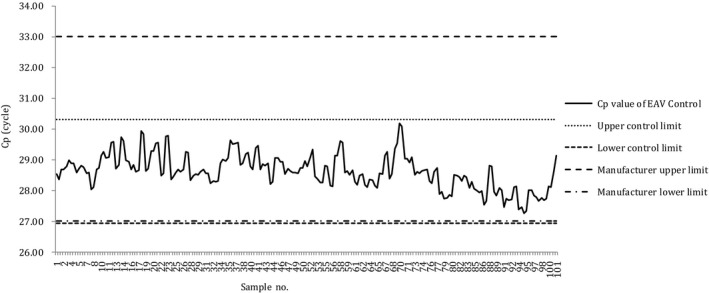
X‐bar control chart of the Cp values of samples processed through the N2 assay performed using automatic nucleic acid extraction. Cp, crossing point; EAV, equine arteritis virus; LCL, lower control limit; UCL, upper control limit

The X‐bar control charts of the Cp values obtained through the N and N2 assays, using the automatic nucleic acid extraction method, are presented in Figures 4 and 5, respectively. In both charts, no cases deviated from the UCL or LCL, and no cases deviated from the recommended range of the EAV reagent.

## 4 DISCUSSION

In this study, the CV values were as low as 0.94–2.02% in both the N and N2 assays, suggesting that the operation/reaction system was stable at all steps of detection when using either the conventional or automatic nucleic acid extraction method. The manual procedure for detecting SARS‐CoV‐2 nucleic acid was performed by seven medical technicians. The tests were stably performed, irrespective of the differences in the technical skills among the technicians. If the CV value increases, it is necessary to take measures, such as confirming the procedure and sample properties and changing the reagents.

In addition, the Cp value of the EAV control was within the range of the UCL and LCL for all the samples processed using either the conventional or automatic nucleic acid extraction method, suggesting that the tests were stably performed.

The CV of the samples processed using the automatic nucleic acid extraction method was larger than that of the samples processed using the conventional method. This could be attributed to the fact that the latter involves RNA extraction, but the former involves total nucleic acid extraction, and the reaction of the EAV reagent is inhibited by the presence of mixed nucleic acids.

In this study, the tests were performed with a limited quantity of reagents and were subject to time constraints owing to the pandemic. It was not possible to collect the EAV reagent data in advance, and therefore, we decided to perform the quality control analysis retrospectively. Ideally, the analytical procedure involves prior data collection, calculation of the UCL and LCL, and generation of test data plots, similar to that in biochemical testing. We have considered adopting this protocol in future. Herein, we investigated a method for achieving quality control in real time. However, the developed method needs to be revised because of the emergence of new SARS‐CoV‐2 variants, such as the alpha, beta, gamma, and delta variants^9^, ^10^, ^11^, ^12^, ^13^, ^14^, ^15^; therefore, developing new technologies for future outbreaks caused by these variants is necessary. In this context, it could be useful to conduct retrospective quality control studies such as this one.

Many manufacturers have developed new SARS‐CoV‐2 detection reagents. Some of these, such as the reagent from Roche Diagnostics, are based on the detection of external controls added to the test samples, whereas others detect the endogenous controls. Irrespective of the control used, the accuracy of the test can be confirmed by plotting a quality control chart; the chart can reveal trends in data fluctuation, thereby enabling resolution of any issues pertaining to the test results.

In addition to the internal quality control, the quality of testing at a facility can be further assured by implementing external quality control, such as participation in the College of American Pathologists surveys.

In conclusion, the calculated EAV control Cp values in our study were within the recommended range for the reagent, and there was almost no deviation from the UCL and LCL. Therefore, in the N and N2 assays described in the Pathogen Detection Manual 2019‐nCoV, the operation and reaction systems at all steps of SARS‐CoV‐2 nucleic acid detection can be considered stable, regardless of the extraction method used. Generating an X‐bar control chart based on the calculated Cp values using quality control substances, such as the EAV reagent, is possible in any laboratory, as long as the kit reagents are published, and the test method is probable. The quality control charts were very useful while performing conventional PCR. The use of such a quality control method could minimize errors in each sample and guarantee the accuracy of the test results. However, this retrospective study was conducted with samples from only one institution and utilized only one detection method. Therefore, to obtain reliable results, future studies should assess data collected from multiple institutions, and on a larger scale.

## Data Availability

The data that support the findings of this study are available from the corresponding author upon reasonable request.
